# A cross-sectional study of Colombian University students’ self-perceived lifestyle

**DOI:** 10.1186/s40064-015-1043-2

**Published:** 2015-06-24

**Authors:** Robinson Ramírez-Vélez, Héctor R Triana-Reina, Hugo A Carrillo, Jeison A Ramos-Sepúlveda, Fernando Rubio, Laura Poches-Franco, Daniela Rincón-Párraga, José F Meneses-Echávez, Jorge E Correa-Bautista

**Affiliations:** Grupo GICAEDS, Facultad de Cultura Física, Deporte y Recreación, Universidad Santo Tomás, Carrera 9 No 51-23, Bogotá, D.C., Colombia; Programa de Licenciatura en Educación Física y Deporte, Universidad del Valle, Meléndez, Cali, Colombia; Facultad Educación a Distancia y Virtual. Institución, Universitaria Antonio José Camacho, Santiago de Cali, Colombia; Centro de Estudios en Medición de la Actividad Física (CEMA), Escuela de medicina y ciencias de la salud (EMCS), Colegio Mayor de Nuestra Señora del Rosario, Bogotá, D.C., Colombia

**Keywords:** Lifestyles, Health promotion, Latin America, Student

## Abstract

**Background:**

The Fantastic Lifestyle Questionnaire was designed for enabling staff working in health sciences and physical activity (PA) areas to measure lifestyles (LS) in the general population. The aim of this study was to assess the lifestyle in a sample of university students.

**Method:**

This was a cross-sectional, descriptive, observational study involving 5,921 subjects’ aged 18- to 30-years-old (3,471 females) from three Colombian cities. Was applied “Fantastic” instrument (that consists of 25 closed items on the lifestyle), translated to Spanish in versions of three and five answers.

**Results:**

Having a “good LS” was perceived by 57.4% of the females and 58.5% of the males; 14.0% of the females rating their LS as being “excellent” and males 19.3% (p < 0.001); 20.3% of the females and 36.6% of the males stated that they spent more than 20 min/day on PA (involving four or more times per week). Negative correlations between FANTASTIC score and weight (*r* = *−*0.113; *p* < 0.01), body mass index (BMI) (*r* = −0.152; *p* < 0.01) and waist circumference (*r* = −0.178, *p* < 0.01) were observed regarding females, whilst the correlation concerning males was (*r* = −0.143, *p* < 0.05) between Fantastic score and weight, (*r* = −0.167 *for BMI*, *p* < 0.01) and (*r* = −0.175, *p* < 0.01 for diastolic blood pressure). In spite of the students being evaluated referring to themselves as having a healthy LS (i.e. giving a self-perceived view of their LS), stated behaviour involving a health risk was observed in the domains concerning nutrition, PA and smoking.

**Conclusion:**

Specific diffusion, education and intervention action is thus suggested for motivating the adoption of healthy LS.

## Background

Lifestyle (LS) is a complex construct which plays a central role concerning the state of an individual’s health (Ramírez-Vélez and Agredo-Zúñiga 2012; World Health Organization 1998). Even though there is no single definition, several authors have suggested that LS is influenced by the habits, attitudes, conduct, traditions, activities and decisions of a person, or a group of people, regarding the various circumstances in which human beings develop in society or during their daily work/career and which are capable of being modified (Ramírez-Vélez and Agredo-Zúñiga 2012; World Health Organization 1998; Flórez Alarcón and Hernandez 1998). The domains making up LS would include behaviour and preferences related to the type of diet/nutrition, physical activity (PA), drinking alcohol, smoking and/or taking other drugs, responsibility for health, recreational activities, interpersonal relationships, sexual practice, work-related activities/career and consumption patterns (López-Carmona et al. 2000).

There is current consensus that LS fundamentally influences the health-disease relationship, meaning that its identification must be a priority for all healthcare professionals, especially those working in health promotion and disease prevention (Ramírez-Vélez and Agredo-Zúñiga 2012). The World Health Organisation (WHO) has stated that a significant percentage of non-communicable diseases (NCD) could be prevented by people modifying their LS and the associated risk factors (World Health Organization 2012), mainly by following a healthy diet and controlling their weight, PA, limiting their alcohol and tobacco intake and keeping their blood pressure and plasma lipid levels within normal limits.

Nevertheless, there is an interaction between LS and genetic, environmental and/or behavioural factors where most NCD are concerned. Regarding LS, it is worth highlighting lifestyles which may be considered modifiable (due to their frequency, magnitude and the possibility of intervention) from a sociocultural and health point of view, such as diet/nutrition, alcohol consumption and smoking, PA and sedentary behaviour. A recent meta-analysis has demonstrated that interventions which have included LS modification may prevent the appearance of NCD in certain types of population group (Souza et al. 2011). Blair et al. (1996) observed that modifying the salt content of young people’s diet/nutrition, consuming fruit and vegetables and regularly engaging in PA reduced cardiovascular risk in most adult stages.

A strong body of research has provided epidemiological evidence of the benefits of having a healthy LS as a protective and independent factor regarding the university population’s physical and mental health (Mogre et al. 2014; González-Jiménez et al. 2013; Tol et al. 2013). Debate focused on ascertaining university students’ LS would thus seem valid, as several studies (Abolfotouh et al. 2007; Abu-Moghli et al. 2010; Tirodimos et al. 2009; Pérusse-Lachance et al. 2010) have emphasised that university life represents a complex stage during which habits and behaviour become determinants throughout the rest of life. Ledo-Varela et al. (2011) and González et al. (2002) have described that the university population as being subject to a series of physiological changes, typical of youth, to which possible sociological and cultural changes may be added due to the start of university studies, leaving home and/or the beginning of adult life. At this stage, students are no longer dependent on their parents and families and therefore assume responsibility for managing their own health. If they are unaware of the importance of health at this stage and continue to engage in risky behaviours such as incorrect eating habits, lack of exercise, smoking, and drinking, the foundations of their health during adulthood may be threatened (Kim et al. 2015). All the foregoing has a direct repercussion on LS.

Nevertheless, few information dealing with Latin-America is available regarding this, especially in Colombia; this means that further studies are needed for investigating this phenomenon in depth (Sanabria-Ferrand et al. 2007; Ramírez-Vélez and Agredo-Zúñiga 2012). The aim of this study was to assess the lifestyle with a sample of Colombian university students.

## Methods

### Settings and study design

A cross-sectional study was carried out during 2013, involving 5,921 healthy subjects aged 18–30 years old from the cities of Bogotá, Cali and Medellín (Colombia). Students were informed that their participation was voluntary with no penalty for not participating. First, the exercise habits of the samples were assessed based on the international PA guidelines (USDHHS). Participants, who perform PA three or more sessions per week at moderate to vigorous intensity for 20 min or above, were classified as active. Remained participants were classified as inactive and perceived barriers to PA of them were evaluated by written questionnaire. If students decided to participate in the study, they gave consent and completed clinical test and two surveys. Questionnaires were completed anonymously, and students were advised that they could terminate their involvement in the study at any time. A convenience sample of at least 6,000 individuals was invited to participate in the study. Subjects having a medical or clinical diagnosis of systemic disease, ≥30 kg m^−1^ BMI were excluded. The students who agreed to participate and who had signed the informed consent form were given appointments for the following procedures:

### Physical exam and clinical variables

Each person was asked to answer questions related to a health survey; their answers regarding sociodemographic data and personal and family pathological background were recorded. Each participant’s height when stretching was recorded using a Krammer anthropometer (Holtain Ltd., Crymych Dyfed, UK) (4 segments, 1 mm precision); their weight was measured by using a Health-o-Meter floor scale (Continental Scale Corp., Bridgeview, III, USA) having 100 g precision, calibrated with known weights according to the protocol described by López-Albán et al. (2008). The BMI was calculated in kg m^−1^ with these variables. A Krammer anthropometric metric tape (Holtain Ltd., Crymych Dyfed, UK) was used for measuring arm, waist and hip circumference. Blood pressure was determined according to Americana Heart Association recommendations (Pickering 1996), using an automatic sphygmomanometer (Omron HEM 757 CAN, Hoofddorp, Holland) on the right arm on two occasions separated by a 5 min interval; the participants were supine and readings taken after 10 min rest. Average blood pressure was calculated using the following formula: (2 × diastolic blood pressure + systolic blood pressure)/3. The aforementioned dimensions and measurements were taken with devices approved by and in line with the International Council of Scientific Unions’ international biological programme standards, covering essential procedures for the biological study of human populations (Weiner and Lourie 1981).

### Measuring lifestyle

Lifestyle was identified by using a version of the FANTASTIC Lifestyle Questionnaire validated for the Colombian population (Ramírez-Vélez and Agredo-Zúñiga 2012); it was used so that each participant could provide a self-perceived concept of their LS. This generic tool was originally designed by the Department of Family Medicine at McMaster University in Canada; this device is considered today to be a valuable tool for aiding healthcare staff working in the area of health promotion and disease prevention as it facilitates identifying and measuring a particular population’s LS (Abu-Moghli et al. 2010). The acronym FANTASTIC represents the first letters of the nine domains in which the 25 questions or items are distributed: F = family and friends; A = activity (physical activity); N = nutrition; T = tobacco & toxics; A = alcohol intake; S = sleep, seatbelts, stress, and safe sex; T = type of behavior (Type A or Type B behavior pattern); I = insight; C = career (work, satisfaction with profession). The questions are distributed on a Likert scale; 23 of them have multiple-choice questions (five answers) and 2 are dichotomous. The alternatives are presented in columns in order to facilitate coding; the left-hand column is always the one with the lowest value or that bears the least relationship with a healthy lifestyle (Rodriguez et al. 2008). Questions are coded by points as follows: 0 for the first column, 1 for the second, 2 for the third, 3 for the fourth, and 4 for the fifth column. For questions with just two alternative answers, the score is 0 for the first column and 4 points for the last column. The sum of all points yields a total score that classifies individuals in five categories, as follows: “Excellent” (85–100 points), “Very good” (70–84 points), “Good” (55–69 points), “Regular” (35–54 points), and “Needing improvement” (0–34 points), (Rodriguez et al. 2008).

### Data analysis

The Statistical Package for the Social Sciences (SPSS-PC) (Chicago, IL, USA, version 21) was used for analysing the results, using univariate analysis for gender and Fantastic domain. A Kolmogorov–Smirnov test was used for determining whether the datasets differed significantly, one tailed analysis of variance (ANOVA) with Tukey’s b post hoc test for continuous variables and Pearson’s correlation coefficient (r) and a linear X^*2*^ test for percentages for determining differences and correlations by gender and FANTASTIC questionnaire internal categories or domains. A logistic regression model was used, calculating the odds ratios (OR) regarding prevalence and 95%CI between each FANTASTIC category and confusion variables, i.e. sex, age and BMI. A *p* < 0.05 value was considered significant.

### Ethical consideration

We took informed consent before conducting the survey and interviews. The ethical review committee of the Universidad del Rosario, reviewed and approved the study protocol.

## Results

The sample consisted of 5921 subjects (3471 female (58.6%) and 2450 male (41.4%). Table 1 gives the participants’ overall characteristics. All parameters evaluated came within the range considered healthy for the equivalent age group.

**Table 1:** The participants’ clinical characteristics and life-style score

Characteristic (mean ± SD)	Female (*n* = 3471)	Male (*n* = 2450)	Total (*n* = 5921)
Age (years)	20.1 ± 2.1	20.6 ± 2.4	20.3 ± 2.1
Weight (kg)	57.3 ± 9.6	67.4 ± 11.2	61.5 ± 11.5
BMI (kg•m^−1^)	22.6 ± 3.4	22.7 ± 3.4	22.6 ± 3.4
Waist circumference (cm)	72.2 ± 8.6	77.7 ± 9.1	74.5 ± 9.2
Hip circumference (cm)	92.4 ± 9.1	92.9 ± 8.5	92.6 ± 8.9
Systolic blood pressure (mmHg)	109.4 ± 12.3	121.1 ± 13.8	114.2 ± 14.2
Diastolic blood pressure (mmHg)	68.8 ± 9.5	70.8 ± 10.5	69.6 ± 9.9
Mean blood pressure (mmHg)	82.3 ± 9.3	87.5 ± 9.7	84.5 ± 2.8
FANTASTIC score	74.0 ± 10.2	75.9 ± 10.5	74.8 ± 10.3

Table 2 describes the differences regarding FANTASTIC score prevalence. The greatest prevalence was found when scoring “good LS” (females 57.4% and males 58.5%), followed by “Excellent LS” (14.0% for females and 19.3% males; p < 0.001). The lowest percentage prevalence by gender was found in the “Regular” category (7.9% females and 6.4% males; p < 0.001). No people were found having scores lower than 34 (and would thereby have indicated “at risk or needing improvement” regarding LS according to the classification scale proposed by FANTASTIC original authors).

**Table 2:** Differences regarding FANTASTIC score prevalence

	FANTASTIC category, *n* (%)				*p* trend (X^**2**^ **)**
	Regular	Good	Very good	Excellent	
Female *n* = 3471 (58.6%)	273 (7.9)	721 (20.8)	1991 (57.4)	486 (14.0)	<0.001
Male *n* = 2450 (41.4%)	158 (6.4)	385 (15.7)	1434 (58.5)	473 (19.3)	<0.001
Total *n* = 5921(100%)	431 (7.3)	1106 (18.7)	3425 (57.8)	959 (16.2)	<0.001

Regarding PA, 20.3% of the females and 36.6% of the males spent 20 min/day on PA and did so four or more times per week. Both groups stated that they “almost always” maintained a balanced diet concerning nutrition (35.3% female vs. 35.6% male). Around 12% of those surveyed referred to “consuming food having high sugar, salt and/or fat content.” 19.0% of the females and 27.1% of the males stated that they “had smoked during the last year.” Significant differences concerning some clinical characteristics according to gender were observed in the general population regarding LS (total FANTASTIC score); differences were observed concerning BMI and diastolic blood pressure in the group of males and overall total (Table 3).

**Table 3:** Relationship between the life-style (FANTASTIC score) and clinical characteristics by gender and general population

Group	FANTASTIC category, *n* (%)				Total score	*p* value
	Regular	Good	Very good	Excellent		
Females						
BMI, kg•m^−1^	23.5 ± 3.8	23.1 ± 3.7	22.4 ± 3.1	22.1 ± 2.8	22.6 ± 3.3	e,f
Waist circumference, cm	74.5 ± 8.9	73.3 ± 9.2	71.6 ± 8.1	70.6 ± 7.7	72.0 ± 8.4	ns
Systolic blood pressure, mmHg	108.3 ± 11.9	109.2 ± 13.1	109.2 ± 12.1	109.9 ± 12.3	109.2 ± 12.3	ns
Diastolic blood pressure, mmHg	69.2 ± 9.1	69.7 ± 9.7	69.0 ± 9.3	68.3 ± 10.2	69.0 ± 9.5	ns
Average blood pressure, mmHg	82.2 ± 8.9	82.9 ± 9.7	82.3 ± 9.2	82.2 ± 9.6	82.4 ± 9.3	ns
Males						
BMI, kg•m^−1^	23.6 ± 3.6	23.2 ± 3.7	22.8 ± 3.2	22.4 ± 2.8	22.9 ± 3.3	a,b
Waist circumference, cm	79.5 ± 10.0	78.6 ± 9.7	77.7 ± 8.8	77.1 ± 7.2	77.9 ± 8.8	b
Systolic blood pressure, mmHg	120.1 ± 13.5	120.0 ± 14.5	121.1 ± 13.8	122.0 ± 13.1	121.1 ± 13.8	ns
Diastolic blood pressure, mmHg	74.5 ± 11.5	71.8 ± 10.4	71.0 ± 10.6	71.1 ± 10.3	71.4 ± 10.6	a,b,c
Blood pressure Media, mmHg	89.7 ± 9.9	87.8 ± 10	87.7 ± 9.8	88.1 ± 9.4	87.9 ± 10.5	ns
Total						
BMI, kg•m^−1^	23.5 ± 3.8	23.1 ± 3.7	22.6 ± 3.1	22.3 ± 2.8	22.7 ± 3.3	a,b
Waist circumference, cm	76.3 ± 9.6	75.1 ± 9.7	74.1 ± 8.9	73.8 ± 8.1	74.4 ± 9.0	ns
Systolic blood pressure, mmHg	112.6 ± 13.7	113.0 ± 14.5	114.2 ± 14.1	115.9 ± 14.1	114.1 ± 14.2	ns
Diastolic blood pressure, mmHg	71.1 ± 10.3	70.4 ± 10.0	69.8 ± 9.9	69.7 ± 10.4	70.0 ± 10.1	ns
Average blood pressure, mmHg	85.0 ± 9.9	84.6 ± 10.1	84.6 ± 9.8	85.1 ± 10.0	84.7 ± 9.9	ns

*ns* not significant

^a^ *p* < 0.05—differences between poor vs. good.

^b^ *p* < 0.05—differences between poor vs. excellent.

^c^ *p* < 0.05—differences between poor vs. acceptable.

^d^ *p* < 0.05—differences between acceptable vs. good.

^e^ *p* < 0.05—differences between acceptable vs. excellent.

^f^ *p* < 0.05—differences between poor vs. acceptable.

Negative correlations between total FANTASTIC score and weight (*r* = −0.113, *p* < 0.01), BMI (*r* = −0.152, *p* < 0.01), waist circumference (*r* = −0.178, *p* < 0.01) and hip circumference (*r* = −0.148, *p* < 0.01) were found in the group of females when stratifying by gender. Negative correlations were observed in males regarding body weight (*r* = −0.143, *p* < 0.05), BMI (*r* = −0.167, *p* < 0.01), hip circumference (*r* = −0.191, *p* < 0.01) and diastolic blood pressure (*r* = −0.175, *p* < 0.01), Table 4.

**Table 4:** Partial correlations between clinical characteristics and total FANTASTIC score

Characteristic	Females (*n* = 3104)	Males (*n* = 2200)	Total (*n* = 5304)
Weight, kg	−0.113**	−0.143*	−0.132*
BMI, kg•m^−1^	−0.152**	−0.167**	−0.181**
Waist circumference, cm	−0.178**	−0.038	−0.158**
Hip circumference, cm	−0.148**	−0.191**	−0.120**
Systolic blood pressure, mmHg	0.023	0.147*	0.169**
Diastolic blood pressure, mmHg	−0.031	−0.175**	−0.240**
Average blood pressure, mmHg	−0.013	−0.032	0.005

* Significant at *p* < 0.05.

** Significant at *p* < 0.01.

The group of females in the simple regression analysis revealed a protective association regarding the c (OR = 0.67, 95%CI, 0.60–0.75), “very good LS”, (OR = 0.54, 95%CI, 0.49–0.61) and “excellent LS” (OR = 0.57, 95%CI, 0.51–0.64). This observation also appeared in the 20 to 23 year-old age group concerning the “good LS” (OR = 0.83, 95%CI, 0.74–0.94) and in those over 23 years old (OR = 0.86, 95%CI, 0.74–0.99) concerning “very good LS”, Table 5.

**Table 5:** Logistic regression by gender, age group and nutritional classification by BMI and FANTASTIC category

Category	Gender	Age		Nutritional classification
	Female ^a^ OR (95%CI)	20 to 23 years ^b^ OR (95%CI)	>23 years ^b^ OR (95%CI)	BMI > 25.1 kg•m^−1 c^ OR (95%CI)
Regular	1.10 (0.95–1.28)	1.01 (0.90–1.14)	0.93 (0.80–1.08)	0.92 (0.80–1.05)
Good	0.67 (0.60–0.75)	0.83 (0.74–0.94)	1.06 (0.91–1.24)	0.88 (0.77–1.01)
Very good	0.54 (0.49–0.61)	0.92 (0.82–1.03)	0.86 (0.74–0.99)	0.87 (0.76–1.12)
Excellent	0.57 (0.51–0.64)	1.06 (0.94–1.19)	1.16 (1.01–1.34)	0.78 (0.69–1.09)

*OR* odds ratio, *95%CI* 95 % confidence interval.

^a^Reference group: males.

^b^Reference group: <20 years old.

^c^Reference group: BMI (18.5–25.0 kg•m^−1^).

## Discussion

This study was conducted to yield baseline data that would ultimately be used to establish health care and basic health management policies for university students. An investigation of the health-promoting lifestyles and cardio-metabolic risk factors of students in Colombia could be further used to develop an appropriate university physician intervention programme for this population.

Regarding whether recommendations for PA were being fulfilled in the population being studied, it was observed that 20.3% of females and 36.6% of males engaged in more than 20 min/day and did so four or more times per week. This finding gave a rate below the 53.5% PA prevalence for 18 to 65 year-old adults regarding fulfilling recommendations made by the Colombian Nutritional Situation Survey (Ministerio de la Protección Social 2010) or the 57% accomplishment of PA recommendations in adults reported for the Americas (Hallal et al. 2012). These results were comparable to those of other studies. In the United States, the reported prevalence of PA is 28.6% (Bauman et al. 2012; Kohl et al. 2012) and European countries are 56.7% (World Health Organization 2011).

Latin America has one of the highest urbanization rates in the world with 80% of the population living in urban centers. Taking into account this context, associations between perceived environmental attributes and physical activity may vary from those observed in high income countries (Arango et al. 2013). For instance, Latin American cities are characterized by high land-mix use as well as higher density and connectivity; these attributes have commonly been found to be associated with physical activity (Arango et al. 2013; O’Donovan et al. 2010; Ramírez-Vélez et al. 2011; World Health Organization 2011; Haskell et al. 2007; Organización de las Naciones Unidas 2011; Filozof et al. 2001; Braguinsky 2002; Kohl et al. 2012; Bauman et al. 2012).

On the other hand, similar studies have investigated the relationship between health and lifestyle factors (i.e. drinking or smoking) among university students (Haug et al 2013; Wicki et al. 2010), most of these studies were conducted in Western countries and the study participants were mostly university students from Iran, Taiwan, Spain, Urugay, Ghana, Canada and Greek (Tol A et al. 2013; Kim et al. 2015; Ledo-Varela et al. 2011; Llambí et al. 2012; Mogre et al. 2014; Pérusse-Lachance et al. 2010; Tirodimos et al. 2009). Furthermore, there is a limited amount of research regarding health-promoting lifestyles among university students in Latin-American countries such as Chile and Colombia (Cervera et al. 2013; Ramírez-Vélez et al. 2011).

In the present study, both groups stated that they “almost always” followed a balanced diet (35.3% females vs. 35.6% males). Regarding such behaviour, a deficit concerning balanced nutrition was found which was reflected in the reply “sometimes” concerning the consumption of fruit and vegetables (females 54.1 vs. 53.6%). Such nutritional pattern coincided with that recently reported by Cervera et al. (2013) and Córdoba et al. (2013) who found that the university student population being studied had a poor quality diet which was characterised by low fruit and vegetable intake (Kushi et al. 2012). According to the World Cancer Research Fund, the importance of high fruit and vegetable intake for health, preventing some micro-nutrient deficiencies and especially regarding NCD has been extensively documented during recent years (Jacoby and Keller 2004). Moreover, the observed reduction regarding fruit and vegetable consumption in the population being studied partly coincided with the so-called “modernisation” of nutritional/dietary patterns and results from rapid urbanisation, increased income and ongoing technological innovation regarding food production, processing and sale (Jacoby and Keller 2004). Around 12% of those surveyed referred to consuming food having high sugar, salt and/or fat content and 17% referred to soda-type drinks. It has been shown that an excessive consumption of energy and nutrients such as sodium, saturated fats, carbonated and fluid-replacement (hydration) beverages increases the long-term risk of developing chronic degenerative diseases, including obesity (Drewnowski and Specter 2004). Hedrick et al. (2012) recently observed that empty calories (433 kcal solid fat and 365 kcal added sugar) account for almost 40% of the energy consumed by young adults in the USA (798 of 2,027 kcal/day) and sugary drink (sodas and sugary fruit drinks) consumption accounts for 173 kcal/day. Nevertheless, it should be noted that choosing food, preparing it and the portions consumed do involve decisions which will depend on each student but that such choice is directly involved in the nutritional status and development of healthy LS which are important for their health in the future health (Córdoba et al. 2013).

Regarding self-perception of body weight evaluated in Item 7 (*I am within _____ kg of my healthy weight?*) it was observed that around 20% of the students stated that they weighed 5 kg or more above their ideal weight. However, this study did not evaluate the relationship between body weight *or* calculating BMI for estimating whether self-perception of being overweight and obesity indicated an altered BMI. When stratifying data by gender, negative correlations were found between total FANTASTIC score and weight (*r* = −0.113, *p* < 0.01), BMI (*r* = −0.152, *p* < 0.01), waist circumference (*r* = −0.178, p < 0.01) and hip circumference (*r* = −0.148, *p* < 0.01) in the group of females. Negative correlations were observed in males regarding body weight (*r* = −0.143, *p* < 0.05), BMI (*r* = −0.167, *p* < 0.01) and hip circumference (*r* = −0.191, *p* < 0.01). Studies involving a Hispanic population have demonstrated poor agreement between self-perception of body image and nutritional state (Mínguez Bernández et al. 2011) and a tendency towards being overweight and obesity in college students (Salcedo et al. 2010; Zaragoza Martí and Ortiz-Moncada 2012) without involving eating disorders (Arroyo et al. 2008).

Concerning practice related to smoking “*during the last year*”, more male smokers were found (27.1% males vs. 19.0% females). This result was lower than that reported by Saldivia and Vizcarra (2012) where smoking prevalence was 48% and close to the 28.1% prevalence reported by Llambí et al. (2009). In spite of domains such as PA, nutrition and smoking having prevalence associated with the risk of suffering an NCD, most university students evaluated in the present study had a score representing healthy LS. For example, the combination of smoking and drinking was often in university male students in a recent Swedish study (Haug et al. 2013) and in review articles among European student populations (Wicki et al. 2010). It was found that many participants needed to manage their smoking and drinking habits. Efforts to minimize tobacco use and harmful alcohol consumption are required to achieve cardiovascular well-being.

Lack of reports involving FANTASTIC in the biomedical literature hampers extrapolating and discussing the present study’s results. A report by Rodríguez et al. (2003) was found which dealt with 412 adults suffering diabetes mellitus type 2 and its association with the instrument’s nine domains; metabolic control variables (lipid profile and glycaemia) had weak correlation with result variables (nutrition with BMI, smoking with TAD and TAS, type of personality and work satisfaction). Triviño et al. (2009) observed inverse correlations in 76 25- to 40-year-old females regarding nutrition, having lower BMI values (*r* = −0.39, *p* < 0.01) and a positive relationship between less smoking and lower systolic blood pressure values (*r* = 0.24, *p* < 0.05). PA was negatively correlated with plasma triglyceride levels (*r* = −0.29, *p* < 0.05) in males (n = 71).

Our evidences of behavioural risk factors in students from Colombia just reflect of the current epidemiologic transition reported by Omran several years ago (Omran 2005). The risky behaviours we found in the present study can be assumed as a multidimensional product caused by the urbanization phenomena and also by several barriers for adopting healthy lifestyles such as the lack of health promotion actions aiming to galvanize people in order to counteract NCD epidemic. As an implication, our results can provide further insight for decision makers about the current situation of the most common modifiable risk factors and to establish priority for public health strategies (i.e., tobacco control, the promotion healthy diets and physically active lifestyles).

Some limitations were evident in this study. First, the current study was conducted at a single university, which limits its generalizability to other population groups. Filling out the FANTASTIC checklist by self-reporting could have led to memory bias. Nevertheless, such limitations did not compromise the results obtained here as they were similar regarding total score by gender and similar to that reported in studies carried out in Colombian (Ramírez-Vélez and Agredo-Zúñiga 2012; Triviño et al. 2009), Canadian (Wilson and Ciliska 1984), Brazilian (Rodriguez et al. 2008) and Mexican populations (López-Carmona et al. 2000) and their content led to evaluating LS found in the population. Sociocultural and economic profiles of the students participated to this study may be different from the students attended to other universities in the Colombia. Nevertheless, the confusion variables most frequently associated with LS were included in the regression models (e.g. sex, age and BMI). Such findings are useful as dependent variables or for determining how to orientate the general population, especially the university population, concerning the benefits for health of adopting active LS. The target population should be extended to different age groups as the FANTASTIC checklist is open to problems inherent in all self-reporting questionnaires (i.e., sensitivity to social prejudice, coexistence and coherence). Further studies should be orientated towards contributing evidence regarding LS concerning other variables concerning physical and mental health and healthy environments and other population samples in Latin populations.

Certain health-promoting habits must thus become modified in the population being evaluated to protect against the future development of some chronic diseases. Healthy LS and behaviour patterns are thus suggested for improving LS and, regarding public health strategies, the FANTASTIC Lifestyle Questionnaire could become a preventative tool concerning NCD factors in university students. In addition, coordination with other educational and health and wellness strategies has produced viable and sustainable interventions. This practice would support the Millenium Development Goals to promote the health of young people in developing countries (WHO 2012).
